# Pyoverdine binding aptamers and label-free electrochemical detection of pseudomonads

**DOI:** 10.3389/fchem.2024.1438710

**Published:** 2024-08-01

**Authors:** Sharif Anisuzzaman, Nima Alimoradi, Dilini Singappuli-Arachchige, Soma Banerjee, Gennady V. Pogorelko, Yunus A. Kaiyum, Philip E. Johnson, Pranav Shrotriya, Marit Nilsen-Hamilton

**Affiliations:** ^1^ Ames Laboratory, U. S. Department of Energy, Ames, IA, United States; ^2^ Roy J. Carver Department of Biochemistry, Biophysics and Molecular Biology, Iowa State University, Ames, IA, United States; ^3^ Department of Mechanical Engineering, Iowa State University, Ames, IA, United States; ^4^ Aptalogic Inc., Ames, IA, United States; ^5^ Department of Chemistry, York University, Toronto, ON, Canada

**Keywords:** NAAO, DNA aptamer, SELEX, pyoverdine, aptasensor

## Abstract

Pyoverdines are iron-chelating siderophores employed by various pseudomonads to promote their growth in iron-limited environments, facilitating both beneficial and detrimental interactions with co-inhabiting microbes or hosts, including plants and animals. The fluorescent pseudomonads produce fluorescent pyoverdines comprised of a conserved central chromophore and a unique strain-specific peptidic side chain produced by non-ribosomal peptide synthetases. Pyoverdine Pf5 (PVD-Pf5) is produced by *Pseudomonas protegens* Pf-5, a species known for supporting plant growth and its involvement in plant pathogen control. To develop a means of exploring the dynamics of *P. protegens* activity in soil and in the rhizosphere, we selected DNA aptamers that specifically recognize PVD-Pf5 with high affinities. Two selected aptamers with only 16% identity in sequence were examined for structure and function. We found evidence that both aptamers form structures in their apo-forms and one aptamer has structural features suggesting the presence of a G-quadruplex. Although their tertiary structures are predicted to be different, both aptamers bind the target PVD-Pf5 with similar affinities and do not bind other siderophores, including the related pyoverdine, pseudobactin, produced by *Pseudomonas* sp. *B10*. One aptamer binds the pyoverdine peptide component and may also interact with the chromophore. This aptamer was integrated into a nanoporous aluminum oxide biosensor and demonstrated to successfully detect PVD-Pf5 and not to detect other siderophores that do not bind to the aptamer when evaluated in solution. This sensor provides a future opportunity to track the locations of *P. protegens* around plant roots and to monitor PVD-Pf5 production and movement through the soil.

## 1 Introduction

The rhizosphere, surrounding the root, is the habitat of a variety of organisms including many microbes. Plants encourage the development of a favorable microbial community by exuding metabolites and proteins that attract, suppress, or kill microbes. The microbial community contributes to the plants’ access to nitrogen and bioavailable minerals (Bais et al., 2006; van der Heijden et al., 2008; Jacoby et al., 2017). Microbes such as pseudomonads can protect plants from pathogenic microbes (Haas and Défago, 2005; Wang et al., 2022) and are frequent rhizosphere residents. Seventy percent of 209 culturable species isolated from maize rhizosphere (∼7 species/plant) were in the *Pseudomonas fluorescens* subgroup (Vacheron et al., 2016). Each pseudomonad species releases a unique pyoverdine (Meyer, 2000), which chelates Fe^3+^ and is taken up by the bacterium by way of cell surface receptors that specifically recognize the pyoverdine of that species. *Pseudomonas protegens,* which releases pyoverdine Pf5 (PVD-Pf5), also expresses multiple outer membrane receptors that recognize pyoverdines from other strains (Hartney et al., 2013). Most plant-microbe interactions are studied in hydroponic systems and provide only end point results. However, plant-microbe interactions are dynamic and there is a need for biosensors to study these interactions in real time. This study focuses on the selection, development, and characterization of aptamers for pyoverdine and the incorporation of one aptamer into an aptamer-based biosensor that will be further developed for measuring pyoverdine in the soil.

Aptamers are single stranded nucleic acids that can be selected by an *in vitro* evolutionary protocol called “Systematic evolution of ligands by exponential enrichment” (SELEX) to bind specific targets including proteins, small molecules, and cells with high affinity and specificity (Ellington and Szostak, 1990; Tuerk and Gold, 1990). Here we describe the selection of two DNA aptamers that bind pyoverdine. To our knowledge this is the first report of DNA aptamers that recognize this siderophore, which plays a prominent role in microbial interactions in the rhizosphere. A computational prediction that one aptamer forms a G-quadruplex was investigated by several biochemical and biophysical methods for both aptamers. One aptamer was found to bind the peptide moiety of pyoverdine and to also have some interaction with the chromophore. When incorporated in a nanoporous aluminum oxide biosensor, this aptamer could selectively detect pyoverdine.

## 2 Results

### 2.1 Aptamer selection

**FIGURE 1:**
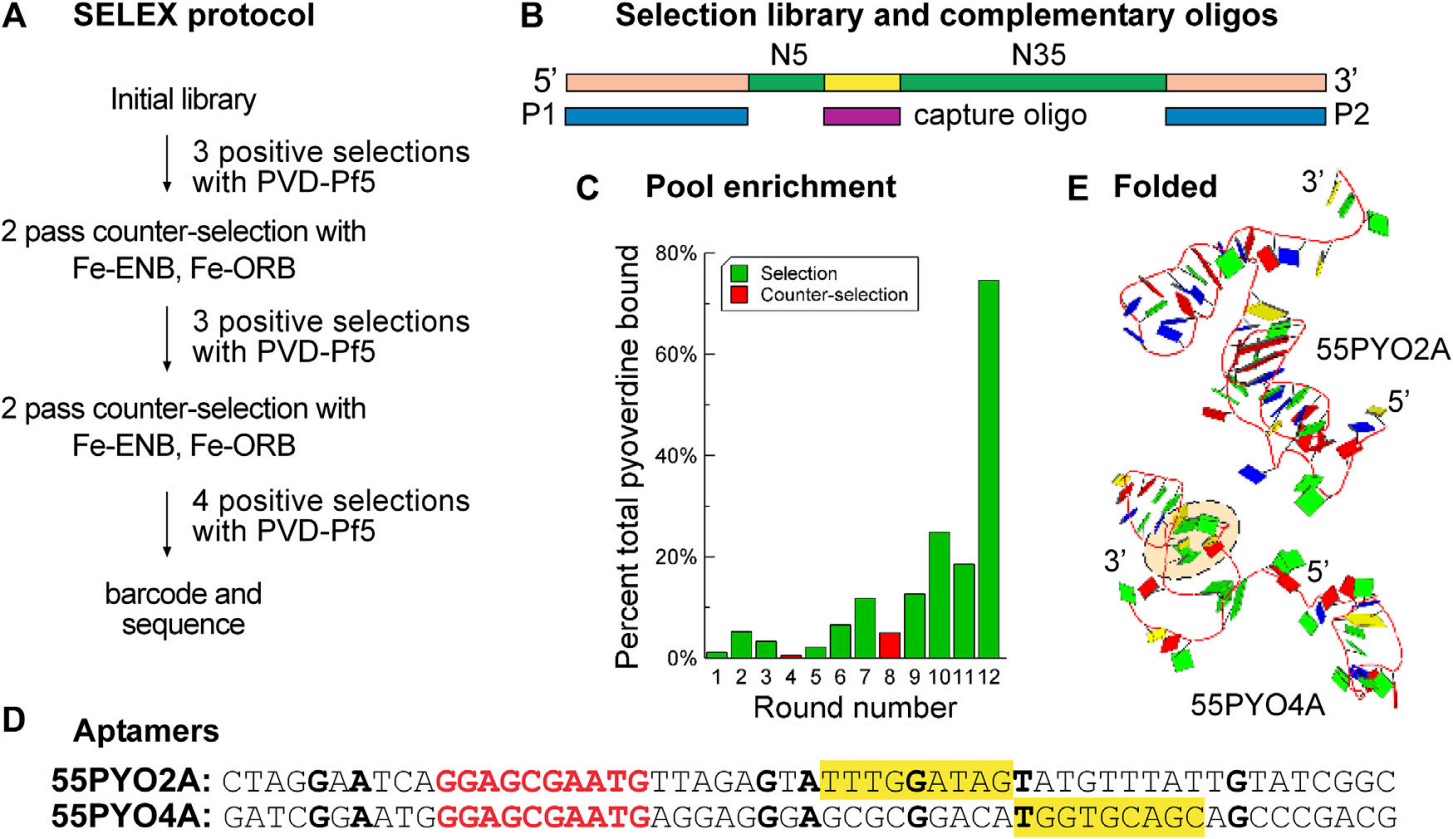
SELEX enriches the nucleic acid population for pyoverdine-binding oligonucleotides. **(A)** Flow diagram for SELEX protocol for selecting aptamers that bind pyoverdine pf5. **(B)** Design of the SELEX library (N10 and N35 (green) are random sequence regions, an additional 10 nt constant region (yellow) is complementary to the capture oligo (maroon), and oligonucleotides P1 and P2 (blue) are complementary to the primer sites (peach). The capture oligo is biotinylated and attached to streptavidin-coated beads for library capture and presentation to pyoverdine. Oligonucleotides released on the addition of pyoverdine are considered to have bound pyoverdine. **(C)** The percent of DNA from each selection (green bars) or counter selection (red bars) round that bound pyoverdine. **(D)** Sequences of selected aptamers 55PYO2A and 55PYO4A. The bolded red text is the 10 nt constant region complementary to the capture oligo sequence. The bolded black bases are those that are in identical positions in both aptamers. The yellow-filled rectangles identify a G-rich motif found in both aptamer clusters. **(E)** Predicted 3D structures of 55PYO2A and 55PYO4A. The dashed peach-filled oval shows a G-quadruplex predicted in the 55PYO4A structure.

Aptamer selection was performed as described in *Materials and Methods* section (Figures 1A, B). This protocol was designed to isolate aptamers with two characteristics. The first characteristic was that the desired aptamers will change their conformations when they bind their targets. This characteristic was identified because a conformational change on binding its target is expected to make an aptamer a more effective sensing unit on a biosensor. To select structure-switching aptamers, the oligonucleotide library sequence contained a centrally located constant region that enabled the library of oligonucleotides to hybridize with the complementary capture oligonucleotide attached to the capture matrix (streptavidin-magnetic beads). We also hybridized the oligonucleotide library with oligonucleotides that were complementary to the primer binding regions located at the ends, which focused the selection pressure on the central randomized region. Two benefits of this strategy were: 1) a streamlined informatics search due to the presence of fewer bases per sequence and 2) ease of experimental screening with options for labeling prospective aptamers with a single labeled oligonucleotide that is complementary to one of the primer binding sequences without danger of interfering with the aptamer structure.

Aptamer selection started with an equimolar ratio of pool to PVD-Pf5 and was continued with a harmonic increase in the ratio of oligonucleotide pool to target PVD-Pf5 in subsequent cycles to increase the selection pressure (Levine and Nilsen-Hamilton, 2007). Counter selection was performed after rounds three and six against a mixture of enterobactin (ENB) and ornibactin (ORB), two other siderophores that are frequently found in soil (Figure 1A). A capture/release approach was used for selection with the inclusion of additional oligonucleotides to hybridize with the terminal PCR primer sites to prevent the inclusion of these constant regions in the folded aptamer structure (Figure 1B). With the assumption that the oligonucleotide complex with PVD-Pf5 is stoichiometric, this selection protocol resulted in 75% of the PVD-Pf5 bound by the DNA pool by round 12 (Figure 1C).

The results of next-generation sequencing for oligonucleotide pools from several rounds, including the final round of each selection, provided the data for identifying potential aptamer sequences. Successful selections were indicated by several criteria (Supplementary Figure S1), including a rise in enriched species with subsequent selection cycles. Sequence clusters were identified with the Aptasuite open-source software and several oligonucleotides from these clusters were evaluated for binding PVD-Pf5 by determining the ability of PVD-Pf5 to displace a FAM-labeled capture oligonucleotide. As this analysis was performed with streptavidin-coated plates (Supplementary Figure S2), the screening might have identified oligonucleotides that bind streptavidin and be displaced from the streptavidin by pyoverdine. To avoid such oligonucleotides, we then evaluated the binding of identified candidates to pyoverdine using the dye displacement assay in the absence of streptavidin. As well, early screening was performed with the full-length oligonucleotides containing the primer sequences that were hybridized with complements during selection and screening. This screening identified aptamer 55PYO2A. We also used a computational approach to screen potential aptamer clusters (increased enrichment with selection rounds) by positing that a good aptamer might have the tendency to form a strong structure such as a G-quadruplex. Consequently, we evaluated sequences for their predicted G-quadruplex formation. From this screen we identified 55PYO4A. Thus, by experimental and computational analysis, two oligonucleotides were identified as potential aptamers for further investigation, which was performed using the sequences of the selected oligonucleotides stripped of their 5′ and 3′ PCR primer binding regions. These two chosen aptamers were named, 55PYO2A and 55PYO4A, according to their lengths (55 nt) and target (pyoverdine) with a unique number followed by A to identify the oligonucleotide as an aptamer (Figures 1D, E).

### 2.2 PVD-Pf5 binding characteristics of the selected aptamers

**FIGURE 2:**
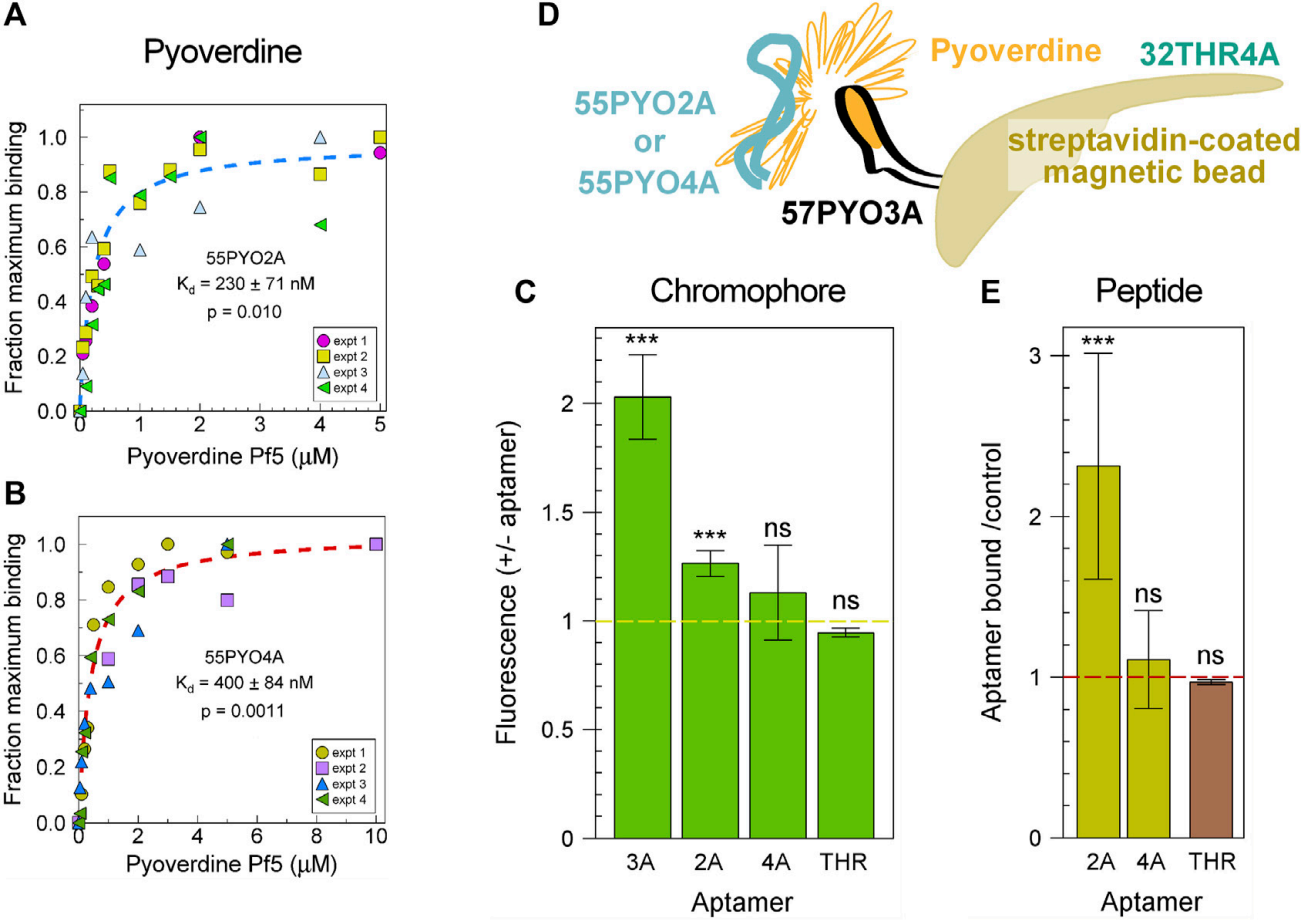
Binding characteristics of the selected pyoverdine-binding aptamers. **(A)** Binding isotherm for 55PYO2A. The data is plotted for four experiments, each performed independently by three individuals. The average of these data was fitted to a Langmuir binding isotherm and is shown as a dashed line. **(B)** Binding isotherm for 55PYO4A. The data is plotted for four experiments, each performed independently by two individuals. The average of these data was fitted to a Langmuir binding isotherm and is shown as a dashed line. The plots in **(A**,**B)** were fit and analyzed for statistical confidence using Sigmaplot. Shown are the estimated *K*_d_, standard deviation, and *p* values. **(C)** Fluorescence changes on binding chromophore for the two aptamers compared with a 2′FY-RNA aptamer that binds the pyoverdine chromophore. The averages of the results from four to six independent experiments are shown as bars with the standard error of the mean as error bars. Asterisks identify when the *p*-value was <0.0005 (***) when fluorescence values with and without aptamer were compared in a paired Ttest. ns = not significant. **(D)** Experimental design to test for aptamer binding to the peptide portion of pyoverdine. The bait, pyoverdine (orange) was bound through its chromophore to 57PYO3A (black), which was linked to streptavidin-coated magnetic beads (brown). ^32^P-labeled aptamers 55PYO2A or 55PYO4A (teal) were the prey. The ^32^P-labeled 32THR4A thrombin aptamer was the control for an effect of pyoverdine on oligonucleotide binding to the streptavidin-magnetic beads (THR). **(E)** The abilities of 55PYO2A and 55PYO4A to bind the pyoverdine peptide presented by 57PYO3A. The cpm (^32^P-55PYO2A or ^32^P-55PYO4A) associated with the streptavidin-coated magnetic beads in the presence of 57PYO3A and pyoverdine is divided by the cpm in the absence of 57PYO3A and pyoverdine (gold bars). The average of five experiments for each DNA aptamer is shown. The effect of pyoverdine on nonspecific binding of DNA to the streptavidin-magnetic beads was tested with ^32^P-32THR4A which was incubated with the streptavidin-beads in the presence or absence of pyoverdine (THR). Shown is the average of results from three experiments in which the cpm of ^32^P-32THR4A bound to the beads in the presence of pyoverdine is divided by the cpm in the absence of pyoverdine (brown). *** = *p* < 0.005.

The binding affinities of the aptamers were determined by a dye displacement assay in which the aptamer, preincubated with thiazole orange, was then incubated with PVD-Pf5 at a range of concentrations. The *K*_d_s were estimated as 230 nM for 55PYO2A (Figure 2A) and 400 nM for 55PYO4A (Figure 2B). As pyoverdine is constituted of two different chemical moieties, the chromophore and a non-ribosomal peptide, we performed two experiments to determine if the aptamers bound the chromophore or the peptide or both. First, we determined if the fluorescence of the chromophore was increased when bound with the aptamer. For this experiment we had a positive control, a previously selected and characterized 2′FY-RNA aptamer, 57PYO3A (Anisuzzaman et al., 2022), that binds and increases the chromophore fluorescence (Figure 2C). When 55PYO2A and 55PYO4A were tested for their effects on chromophore fluorescence, we observed a small but statistically significant increase in fluorescence in the presence of 55PYO2A but no demonstrable increase in fluorescence in the presence of 55PYO4A (Figure 2C). The thrombin aptamer control also did not increase pyoverdine chromophore fluorescence.

To determine if either DNA aptamer bound the peptide portion of pyoverdine, biotinylated 57PYO3A was incubated with streptavidin-coated magnetic beads and then incubated with pyoverdine. We have found that 57PYO3A binds the pyoverdine chromophore. Therefore, when bound by 57PYO3A, some or most of the peptide moiety of pyoverdine is expected to be available to 55PYO2A or 55PYO4A for binding. The magnetic beads with attached 57PYO3A and pyoverdine were incubated with 55PYO2A or 55PYO4A, each 5′-labeled with ^32^P (Figure 2D). The control magnetic beads did not have 57PYO3A attached but they were incubated with pyoverdine. The beads were then washed and evaluated by scintillation counting. The results showed that more ^32^P-55PYO2A remained bound to the PVD-Pf5-57PYO3A-beads after washing compared to the control beads that lacked 57PYO3A and pyoverdine (Figure 2E, gold bars). ^32^P-55PYO4A showed no increase in the amount bound over the control. The thrombin aptamer (^32^P-32THR4A) was used as a control for the effect of pyoverdine on the nonspecific binding of an oligonucleotide to the streptavidin-magnetic beads. The ratio of amount of radiolabeled thrombin aptamer bound to the streptavidin magnetic beads in the presence vs. the absence of pyoverdine (brown bar) shows that pyoverdine does not affect oligonucleotide binding to the beads.

The results of these two studies suggest that 55PYO2A binds the PVD-Pf5 peptide and may interact also with the chromophore. As we saw no evidence of 55PYO4A binding to either the chromophore or to the peptide independently, the interaction of this aptamer with pyoverdine may be more complex and involve both chromophore and peptide in a complex that cannot be formed when the chromophore is bound by another aptamer such as in the configuration used here to test for peptide binding.

### 2.3 Structural features of the PVD-Pf5 aptamers

**FIGURE 3:**
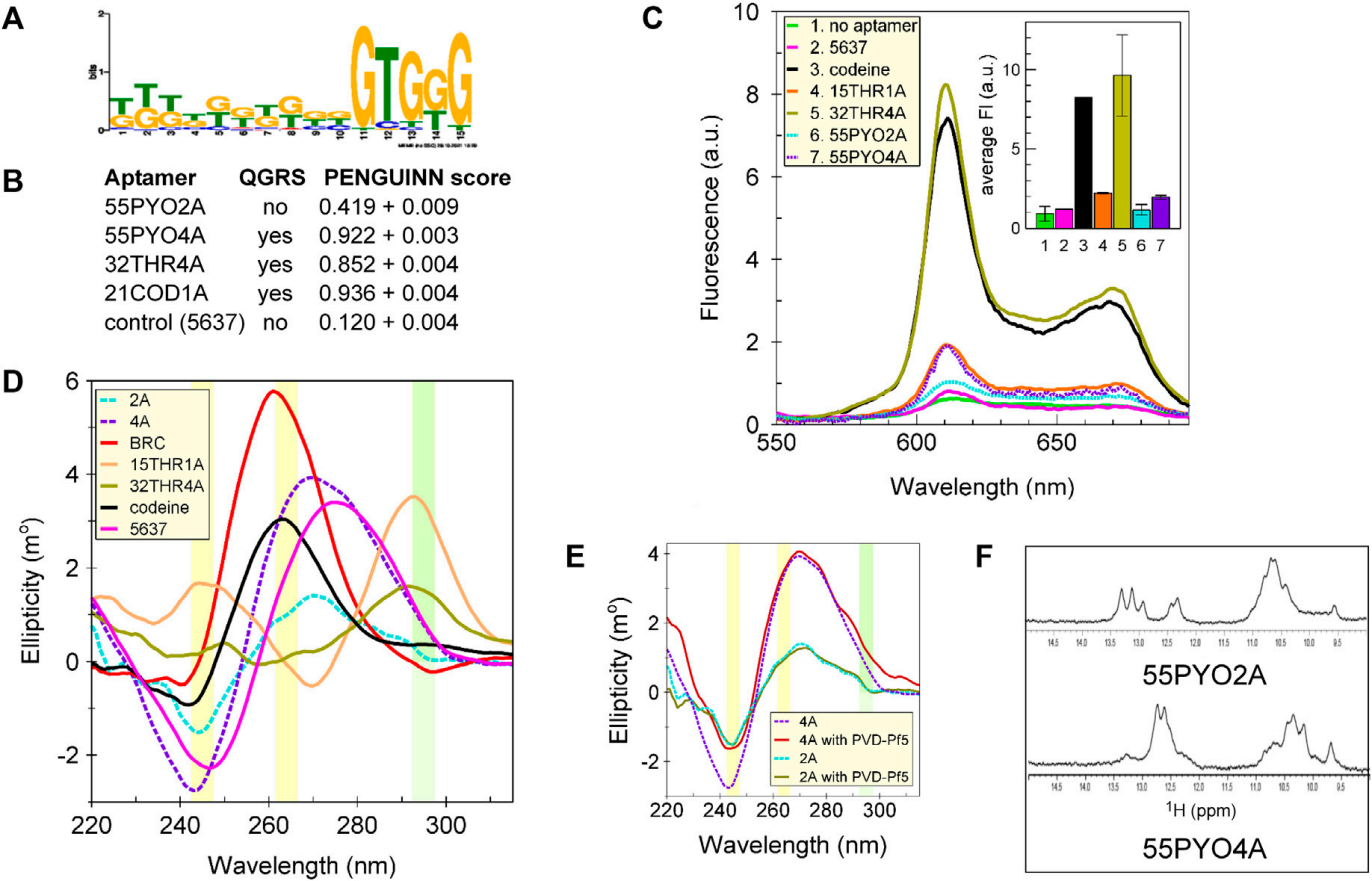
Sequence motif identification and testing for G-quadruplex structure. **(A)** Motif analysis using the MEME suite of the sequences of the top 10K clusters (by size) from SELEX, which included the clusters containing both aptamers, showed G-rich sequences in both aptamers and a common G-rich motif, **(B)** Predictions of a G-quadruplex by QGRS and PENGUINN (accessed 8/30/2022). **(C)** Fluorescence spectra showing the extents of interaction of each aptamer with NMM. **(D)** CD spectra of 55PYO2A, 55PYO4A, Broccoli, 15THR1A, 32THR4A, and Codeine aptamers and oligo 5637 (control oligonucleotide). **(E)** CD Spectra of 55PYO2A and 55PYO4A incubated with and without PVD-Pf5 for 10–30 min depending on the experiment. Each smoothed spectrum is the average of two independently acquired spectra. **(F)** 1D ^1^H-NMR spectra of 55PYO2A and 55PYO4A.

Using the MEME suite (https://meme-suite.org/meme/tools/meme), a motif search was performed on the top ten thousand clusters (by population size), which included those from which the aptamers were chosen. The identified G-rich structural motif (Figure 3A) suggested the possibility of G-quartets in these oligonucleotides. The hypothesis that either aptamer might fold as a G-quadruplex was tested using two prediction algorithms (QGRS and the PENGUINN neural network). Consistent predictions were obtained from both QGRS, which gives a yes/no answer, and PENGUINN, which provides a score to indicate a probability of the presence of a G-quadruplex (Bailey et al., 2015; Klimentova et al., 2020). Whereas 55PYO2A was predicted not to form a G-quadruplex, 55PYO4A was predicted to have a high probability of forming a G-quadruplex (Figure 3B). Tertiary structures predicted using 3dRNA/DNA (http://biophy.hust.edu.cn/new/3dRNA/create) also suggested the presence of a potential G-quadruplex in 55PYO4A but not in 55PYO2A (Figure 1E).

To investigate the structural predictions, we performed three tests for established signatures of G-quadruplexes. First, the aptamers were incubated with N-methyl mesoporphyrin IX (NMM), which selectively binds to G-quadruplexes (Yett et al., 2019). Binding was compared with positive control aptamers that are known to contain G-quadruplexes (two thrombin aptamers and the codeine aptamer) and a negative control oligonucleotide (Oligo 5637) which was not predicted to form a G-quadruplex, and which demonstrated no increased fluorescence over the control condition in the absence of oligonucleotide (Figure 3C). The results for fluorescence in the presence of NMM showed no difference in the fluorescence of 55PYO2A compared with Oligo 5637 but an increase in the fluorescence of 55PYO4A similar to that produced with 15THR1A, which folds to form an antiparallel G-quadruplex (Macaya et al., 1993; Padmanabhan et al., 1993; Wang et al., 1993; Hianik et al., 2007).

In a second test for G-quadruplex structures, we examined the CD spectra of 55PYO2A and 55PYO4A compared with those of the Oligo 5637 and the Codeine, Broccoli, and Thrombin (15THR1A, 32THR4A) aptamers. A parallel G-quadruplex (Codeine and Broccoli aptamers) is characterized by a positive peak around at 264 nm and a weaker negative peak at 242 nm (Nagatoishi et al., 2007), whereas an antiparallel G-quadruplex (Thrombin aptamers) exhibits a peak at 295 nm. The spectra of both 55PYO2A and 55PYO4A are distinct from the parallel and antiparallel G-quadruplexes and do not coincide with the presumed nonstructured Oligo 5637 (Figure 3D). Their peak ellipticities are midway between the known G-quadruplexes and an unstructured oligonucleotide. Considering the possibility that the CD spectra for 55PYO2A and 55PYO4A might reflect their existence as ensembles of structures that include G-quadruplexes and that the distribution of structures might be shifted upon binding the ligand, we incubated the aptamers with pyoverdine and again determined their CD spectra (Figure 3E). The presence of pyoverdine did not alter the CD spectrum of either aptamer.

To further investigate the likely structures of the two pyoverdine aptamers, we acquired their 1D ^1^H NMR spectra. The imino region of the ^1^H spectrum of DNA aptamers, which is diagnostic of their structure (Churcher and Johnson, 2020), is shown in Figure 3F for both 55PYO2A and 55PYO4A. The presence of signals in the 10–12 ppm region is consistent with the formation of non-Watson-Crick base pairs in the DNA structure, which is consistent with a G-quadruplex but may also reflect other structures (Bedrat et al., 2016).

The NMR data, together with the NMM binding and CD spectra suggest that the aptamers are structured in their apo-forms. The possibility that 55PYO4A can adopt a G-quadruplex structure is strengthened by the fact that it binds NMM with the same fluorescence output as observed with the 15 nt thrombin aptamer (15THR1A) for which the presence of a G-quadruplex has been demonstrated by both NMR and Xray crystallography (Macaya et al., 1993; Padmanabhan et al., 1993; Wang et al., 1993).

### 2.4 Aptamer function as the sensing component of a biosensor

**FIGURE 4:**
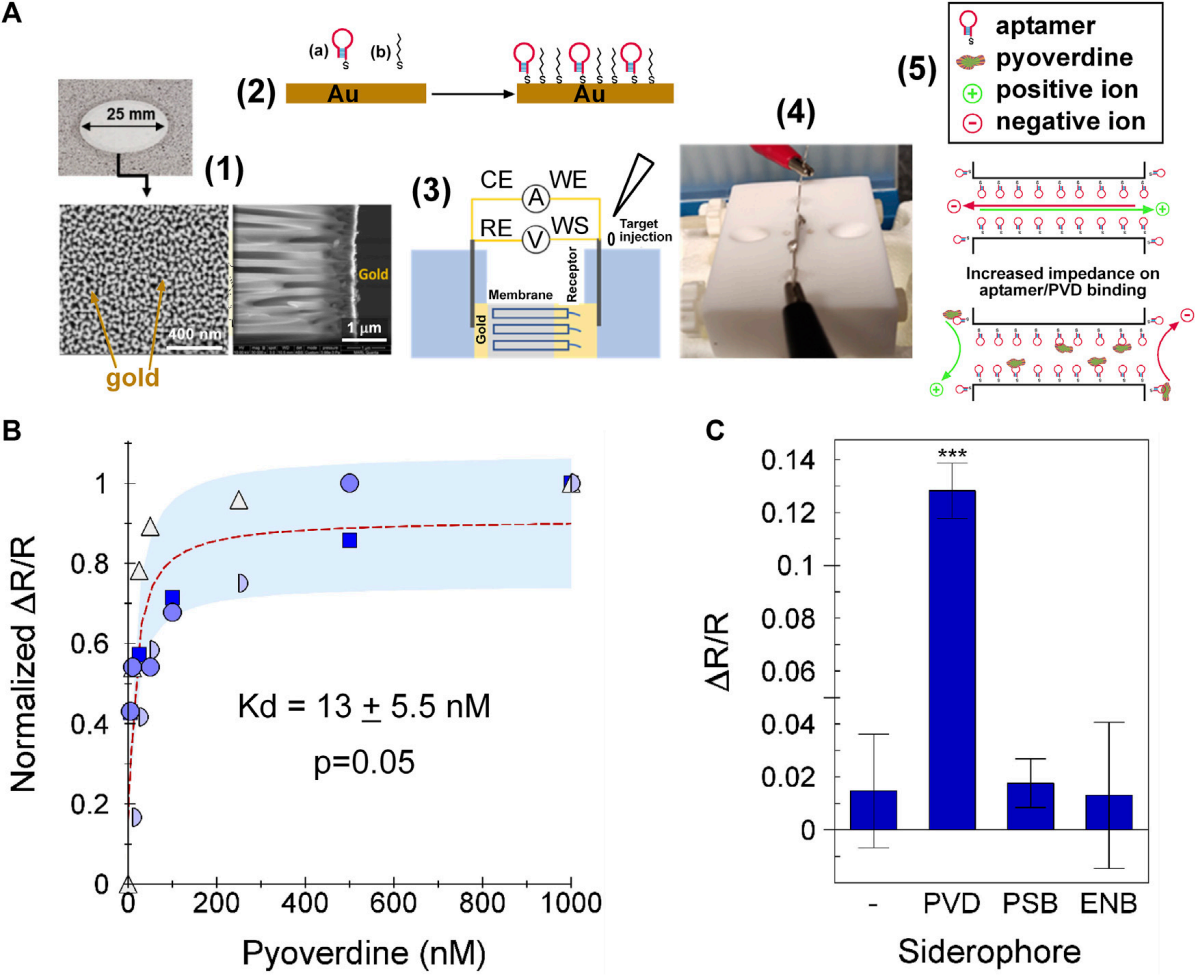
Specificity and function on PVD-Pf5 NAAO sensor. The 55PYO2A-NAAO sensor was prepared as described in Materials and Methods. **(A)** Sensing Flowchart: **(1)** Gold-coated NAAO membrane with details of the 20 nm pore side, **(2)** Functionalization of the gold surfaces with aptamer **(A)** and hexane-thiol **(B)**, **(3)** Schematic and **(4)** photograph of the electrochemical cell with the membrane separating the two chambers, and **(5)** Schematic representation of the impedance changes in the aptamer functionalized membrane on exposure to pyoverdine. **(B)** The sensor was exposed to the identified concentrations of PVD-Pf5, enterobactin or pseudobactin and ΔR/R calculated after subtracting the background. The four input data sets were normalized to the maximum ΔR/R value for each set, which was obtained with 500 nM PVD-Pf5 for one dataset and 1 µM PVD-Pf5 for the remaining three datasets. Each dataset is plotted with a different symbol. The dotted red line is the Langmuir fit for the average of all experiments and the pale blue shaded area is identifies the average one standard deviation from the fit-line, **(C)** The average ΔR/R obtained from each of two experiments with 1 µM pyoverdine Pf5 (PVD), pseudobactin (PSB), or enterobactin (ENB). Error bars show the standard deviation. *** = *p* < 0.001.

We tested the ability of 55PYO2A to detect pyoverdine when incorporated as a sensing element in an electrochemical sensor (Figure 4A). The combined results of four independently performed experiments gave a dynamic range of 3 log units with an estimated *K*_d_ value of 13 ± 5.5 nM (Figure 4B). The results for specificity show that the biosensor could detect pyoverdine with high sensitivity and did not bind either enterobactin or pseudobactin (Figure 4C). Based on a non-linear fit of the normalized data, the limit of detection (LOD) of the sensor was determined to be 1.3 nM and the limit of quantification (LOQ) was 2.2 nM. For comparison, other means reported for detecting pyoverdine are shown in the Supplementary Table S1.

## 3 Discussion

The rhizosphere is an active hub of plant-microbe communications in which pyoverdine fulfills an essential role of iron capture, with its concentration in the soil reflecting the amount of iron available to the community. Each pyoverdine is specific for a pseudomonad species and can be used to identify the activity of that species. PVD-Pf5, a product of *P. protegens*, has the characteristic dihydroxyquinoline chromophore and a unique peptide of eight amino acids synthesized by a series of non-ribosomal peptide synthetases (Loper et al., 2007; Hartney et al., 2013).

Studies of activities in the soil involve the periodic collection of samples by means that are usually destructive. As a result, we know little of the dynamics of interspecies communication in the rhizosphere. To build a sensing element that could be employed below ground to monitor pyoverdine production, we selected DNA aptamers that selectively bind pyoverdine. The primary sequences of these aptamers are quite different with an identity of only 16%.

A G-rich motif was found in the clusters from which these aptamers were derived and so we explored the possibility that the pyoverdine aptamer structures included a G-quadruplex. Analysis of the aptamer sequences by two algorithms designed to identify G-quadruplexes predicted that 55PYO4A, but not 55PYO2A was likely to form a G-quadruplex structure. Therefore, we tested the possibility of a G-quadruplex structure in these aptamers by determining their CD and 1D ^1^H-NMR spectra and if they bind NMM, a molecule with a similar planar structure to a G-quartet that readily slides between two quartets in a G-quadruplex. The results of these studies are consistent with the conclusion that both these aptamers form structures in the apo-form (sans pyoverdine) that can be detected by NMR. As is likely with many short nucleic acids, these aptamers might exist as an ensemble of structures. The ability of 55PYO4A to bind NMM suggests that it might form a G-quadruplex.

The aptamer affinities in solution for pyoverdine are in the range of 200–400 nM and our results suggest that 55PYO2A binds the peptide and perhaps also the chromophore. We were unable to find evidence of which moietie(s) 55PYO4A binds on pyoverdine. Further studies to develop an aptasensor were performed with 55PYO2A, which was better characterized with respect to its interaction with pyoverdine and has a higher affinity for pyoverdine than 55PYO4A.

Specificity for target is essential for a biosensor sensing element. Thus, during selection we performed counter selection against two abundant soil siderophores, enterobactin and ornibactin. Even closer in structure to PVD-Pf5 is pseudobactin, which also contains a dihydroxyquinoline chromophore. Application of 55PYO2A as sensing element to an electrochemical biosensor based on a nanoporous anodized aluminum oxide (NAAO) surface produced a sensor response to PVD-Pf5 but not to enterobactin or pseudobactin, thus demonstrating specificity of the aptamer. The apparent *K*_d_ value determined for this aptamer on the sensor surface was 13 nM, which is much lower than the 230 nM determined for the *K*_d_ in solution. One contribution to the difference in the measured affinities may be that the in-solution assay was a competition assay with thiazole orange, which would lower the observed *K*_d_. However, we and others have consistently observed increases in aptamer affinities when they are attached to a surface compared with when they are free in solution (Zhai et al., 2012; Daniel et al., 2013; Banerjee et al., 2023).

In summary, these studies have identified aptamers that interact specifically with pyoverdine, one of which has been demonstrated to function in the selective detection of pyoverdine as the sensing element in an electrochemical aptasensor. Further development of this aptasensor is directed toward creating a sensor that can operate from below the soil to detect pyoverdine as a reflection of the metabolic activities and interactions of pseudomonads with plants and other microbes in the complex environment of the rhizosphere.

## 4 Materials and methods

### 4.1 Buffers

Synthetic soil salt mix A2 (SSMA2) [366 µM CaCl_2_, 3 µM CuSO_4_, 5 µM MnSO_4_, 27 µM KI, 83 µM KH_2_PO_4_, 35 µM KOH, 1 µM ZnSO_4_, 103 µM Fe (III)Na-EDTA, 68 µM MgSO_4_, 23 μM Mg (NO_3_)_2_, 94 μM MgCl_2_, 64 µM NaCl, 75 µM NH_4_OAc, pH 5.9] was used for aptamer selection and Synthetic soil salt mix B1 (SSMB1) (SSMA2 plus 2 mM MgSO_4_, 10 mM KCl, 1 µM H_3_BO_3_, pH 5.9) as used for analysis unless otherwise specified.

### 4.2 Nucleic acids

All DNAs used in this study were synthesized by Integrated DNA technology (IDT, Coralville, IA). A list of all nucleic acids and their sequences that were used in this study is included in the Supplementary Tables S2, S3. All chemically modified oligonucleotides were purified using HPLC whereas unmodified oligonucleotide sequences were purified using standard desalting unless otherwise stated.

### 4.3 Siderophores

PVD-Pf5, enterobactin and ornibactin were purchased from EMC microcollections, Germany. The structure of PVD-Pf5 is shown in Supplementary Figure S3. Pseudobactin, isolated from *P. fluorescens,* was purchased from Sigma Aldrich (Saint Louis, MO, United States). All the siderophore preparations were in the iron-loaded form.

### 4.4 SELEX library construction

A single stranded DNA library was used (IDT, Coralville, IA, United States) with each oligonucleotide having a length of 100 bases, which includes three constant regions and two random regions: 5′-GCCTGTTGTGAGCCTCCTGTCGAAN (10N) GGAGCGAATGN (35N) GAG​CGT​TTA​TTC​TTG​TCT​CCC-3′ (Figure 1B). In this library N represents an equimolar combination of A, T, C and G. The 5′ and 3′ ends of the constant regions were primer binding sites for PCR amplification and the 10 base long constant region between 10N and 35N was used to capture the ssDNA library components during selection. Specific information regarding the SELEX conditions, oligonucleotide library and complementary oligonucleotides and outcomes are found in Supplementary Table S4; Figure 1 and discussed in the *Results* section. An overview of the NextGEN sequencing data is shown in Supplementary Figure S1) and the full NextGEN sequencing database has been deposited for public access (see Data Availability Statement).

### 4.5 *In vitro* selection of aptamers for PVD-Pf5

SELEX was performed with PVD-Pf5 as target to select DNA aptamers. For the first round of selection a library of 10^15^ ssDNA was used. The biotinylated capture oligos, which are complementary to the short constant region in the library sequence, were attached to streptavidin coated magnetic beads. The DNA library was first hybridized with oligos 5625 and 5617 (complementary to the PCR primer regions) and then immobilized on the beads by hybridization with the capture oligos. The length of the capture oligo varied with the SELEX round as did the hybridization temperature and the oligo:PVD-Pf5 ratio (Supplementary Table S4). The bound oligos were captured using a magnet and purified by ethanol precipitation in the presence of 25 μg/μL linear polyacrylamide (LPA) (Sigma-Aldrich, St. Louis, MO, United States). The ratio of ssDNA to PVD-Pf5 and the length of capture oligo was increased in successive SELEX rounds to increase selection pressure (Supplementary Table S4). Ten rounds of positive selection and two rounds of counter selection were performed (Figure 1C). The counter selections involved two sequential incubations of the bead-captured pool with enterobactin and ornibactin, each at a ratio of 1:1 with the ssDNA pool. All target siderophores used in selection and counter-selection were in the Fe^3+^-bound forms. Synthetic soil salt mix (SSMA2) was used during selection. PCR-amplified libraries were prepared for NGS using Nextera Illumina sequencing primers and the appropriate bar coding. Next-generation sequencing was performed by the Iowa State University DNA facility (HiSeq 3000 platform) and Novogene facility, Sacramento, California using HiSeq PE150 platform.

### 4.6 PCR amplification and ssDNA separation

At the end of each selection round, ssDNAs were amplified over 12 cycles with primers Oligo 5615 and 5’ biotinylated Oligo 5617 with a low fidelity Taq polymerase (Genscript, Piscataway, NJ, United States) and purified using a Qiagen PCR purification kit (Qiagen, Germantown, MD). The dsDNA was incubated with streptavidin-coated magnetic beads (Dynabeads M-280, Thermofisher, Waltham, MA, United States) for 1 h with hybridization buffer (5 mM Tris-HCl, 0.5 mM EDTA, 1.0 M NaCl, pH 7.2) and washed three times with hybridization buffer. The desired nonbiotinylated strand was separated by incubating with freshly prepared 100 mM NaOH for 15 min and neutralized with 100 mM HCl. The ssDNA was purified by ethanol precipitation with the inclusion of 25 μg/μL LPA.

### 4.7 Bioinformatics analysis and data preprocessing

The raw NGS data was demultiplexed and assigned to respective rounds by the open-source software AptaSuite (Hoinka et al., 2018). Barcodes were used to assign DNA pools from different rounds of selection. The existence of no mismatches between the reference and matched barcodes or between the reference and matched primers were criteria for demultiplexing. Further analysis was done with the sequences that met these standards. Clustering was done with 10 iterations of locality sensitive hashing, sampling 55% of the indices in the randomized regions, and allowing no more than 10 nucleotide mismatches between the seed sequence of a cluster to each remaining member. Clusters were extracted for further analysis and aptamer screening. MEME was run with version 5.4.1 using default parameters to analyze the selected sequences derived from the randomized regions in the library (without the primer sequence complements). Secondary structures were predicted using Unafold version 3.6 (Bailey et al., 2015) in the DNA mode and parameters set at 19 mM NaCl, 3 mM Mg^2+^, and 23°C. The probability of G-quadruplex formation was analyzed by the neural network PENGUINN (Klimentova et al., 2020). Tertiary structures of DNA were predicted by 3dRNA/DNA (Wang et al., 2019).

### 4.8 Fluorescence binding assay

Fluorescence emissions by the pyoverdine chromophore were measured by a Cary eclipse fluorimeter (Varian, Palo Alto, CA, United States) and a Synergy 2 (Biotek, Winooski, VT, United States) plate reader. Fluorescence intensities were measured with excitation at λ_ex_ = 400 nm, λ_em_ = 460 nm, and 5 nm slit widths.

### 4.9 Binding with N-methyl mesoporphyrin IX

Two µM each of aptamer and N-methyl mesoporphyrin IX (NMM) from Santa Cruz Biotechnology Inc (Dallas, TX, United States) in SSMB1 were incubated for 10 min at 23°C. Fluorescence was measured at λ_ex_ = 399 nm, λ_em_ = 410–700 nm, slit = 5 nm PMT+800 V using a Cary eclipse fluorimeter.

### 4.10 Circular dichroism spectroscopy

Two µM each of aptamer and PVD-Pf5 in SSMB1 were incubated for 20 min then evaluated for circular dichroism using an MOS-500 monochromator, an ALX-250 light source (Bio-Logic Science Instruments, Grenoble, FR), and a quartz cell (pathlength 1 mm). The spectra were obtained through the accumulation of three scans to average the spectra from 200 to 320 nm. Baselines were recorded for SSMB1.

### 4.11 Dye displacement assay

Twenty nM aptamer in SSMB1 with 1xSYBR gold or 1 µM thiazole orange was heated at 95°C for 5 min and incubated at 23°C for 1 h to allow formation of the dye-aptamer complex. PVD-Pf5 was added to the mixture at a range of final concentrations (25–1,000 nM) and the mixture was incubated for 30 min. The fluorescence difference from the control with no PVD-Pf5 present was measured at λ_ex_ = 485 nm λ_em_ = 496–600 nm at 800 V with a 5 nm slit width. The 535 nm emission was used to calculate the binding affinity.

### 4.12 NMR spectroscopy

NMR experiments on aptamer samples were performed using a 600 MHz Bruker Avance spectrometer equipped with a ^1^H-^13^C-^31^P triple-resonance probe. All NMR spectra were acquired in 10 mM sodium phosphate (pH 6.3) buffer with 0.4 mM CaCl_2_, 2 mM MgCl_2_ and 10 mM KCl in ^1^H_2_O/^2^H_2_O (90%/10%) at 5°C. Water suppression was achieved using excitation sculpting (Hwang and Shaka, 1995). Aptamer concentrations for NMR studies varied between 0.5 and 1.7 mM.

### 4.13 The PVD-Pf5 NAAO sensor and electrochemical impedance spectroscopy

The NAAO sensor consisted of a two-sided gold-coated (by Denton E-beam deposition) Whatman Anodisc nanoporous anodized aluminum oxide (Sigma Aldrich CAT# 6809-6002, St. Louis, MO, United States) to which 5’ thiolated 55PYO2A was attached to both sides followed by 1,6-mercaptohexanol to fill the remaining space on the membrane surface (Gosai et al., 2019). Electrochemical impedance spectroscopy (EIS) scans were collected with the siderophore diluted in SSMB1 in a custom-made Teflon cell. Impedance changes across the Au-NAAO membrane were measured with gold coating on the front and back surfaces of the membrane arranged in a two-electrode configuration. The working electrode/working sense (WE/WS) and counter electrode/reference electrodes (CE/RE) were attached to opposite sides of the membrane using silver wires (Sigma-Aldrich, St. Louis, MO, United States) fused to the gold membrane with silver epoxy (MG chemicals Ltd., Burlington, Ontario, CA) and only the gold coatings were exposed to the electrolyte. Starting with a volume of 1 mL SSMB1, the open circuit potential was measured until it stabilized then sequential additions of siderophore in 20 µL were added up to a maximum cell volume of 1.12 mL, thereby increasing the siderophore concentration in the cell while keeping all other buffer component concentrations unchanged. Three EIS scans were taken after each addition with an AC perturbation signal of 5 mV and over the frequency range of 1 Hz–100 kHz. Averages were taken of the last two scans and analyzed using the Distribution of Relaxation Times (DRT) approach (Wan et al., 2015) to determine the relaxation time spectra with a MATLAB code provided by the Ciucci lab, Hongkong University of Science and Technology (Wan et al., 2015). The parameters used for the DRT analysis included a shape factor coefficient of 0.5, regularization parameter of 10^–3^, and derivative of the 2^nd^ order.

### 4.14 Statistical analysis

All experiments were performed in triplicate and error bars for standard deviation are shown in the figures. Error bars represent standard deviations (SD) calculated as SD = $\sqrt{{Se}^{2}+{Sb}^{2}}$ where *Se* = standard deviation of the sample and *Sb* = standard deviation of the subtracted blank. All binding isotherms were fit to the Langmuir equation [A = (A_max_ ∗ L)/(L + *K*_d_)] where A = the measure of absorbance or fluorescence with A_max_ being the maximum value, L = ligand concentration, and *K*_d_ is the dissociation constant. Fittings and estimations of statistical significance were performed using Sigmaplot. All reported values for *K*_d_ passed the Normality (Shapiro-Wilk) and the Constant Variance (Spearman Rank Correlation) tests.

## Data Availability

The datasets presented in this study can be found in online repositories. The names of the repository/repositories and accession number(s) can be found below: https://www.ncbi.nlm.nih.gov/geo/, GSE225440, https://www.ncbi.nlm.nih.gov/geo/, GSM7048674.

## Abbreviations

15THR1A, 15mer thrombin aptamer; 32THR4A, 32mer thrombin aptamer; 55PYO2A, 55mer ssDNA aptamer reported here; 55PYO4A, 55mer ssDNA aptamer reported here; BRC, Broccoli aptamer; COD, codeine aptamer; ENB, enterobactin; ORB, ornibactin; PVD-Pf5, pyoverdine Pf5; PSB, pseudobactin.
