# Supplementary material for: Pyoverdine binding aptamers and label-free electrochemical detection of pseudomonads
Source: Front Chem. 2024 Aug 1;12:1438710. doi: 10.3389/fchem.2024.1438710 (PMC11324436; doi:10.3389/fchem.2024.1438710)
Supplement: Supplementary file 1 [file DataSheet1.pdf]

Supplementary Material

Pyoverdine Binding Aptamers and Label-Free Electrochemical Detection of Pseudomonads

Sharif Anisuzzaman,[1,2] Nima Alimoradi,[3] Dilini Singappuli-Arachchige,[1,4] Soma Banerjee,[4] Gennady V. Pogorelko,[1,4] Yunus A. Kaiyum,[5] Philip E Johnson,[5] Pranav Shrotriya,[1,3] Marit Nilsen-Hamilton[1,2]

[1]Ames Laboratory, U. S. Department of Energy, Ames, Iowa; [2]Roy J. Carver Department of Biochemistry, Biophysics and Molecular Biology, Iowa State University, Ames, Iowa; Iowa; [3]Department of Mechanical Engineering, Iowa State University, [4]Ames, Iowa; Aptalogic Inc., Ames, IA; [5]Department of Chemistry, York University, Ontario, Canada

TABLES

Table S1. Comparison of methods for detecting pyoverdine

Method	Detection method	sensitivity	detection limits
**Colorimetric**	Binding of pyoverdine and other siderophores to iron results in a color change (Louden et al., 2011)	Positive for all siderophores	Visual indicator, and quantification could be challenging
**Fluorescence Assays**	Intrinsic fluorescence of chromogenic metabolite (Cueva et al., 2020)	Linearity range of 10-1000 ng/ml (7.3 nM – 732 nM)	LOD<br>0.9 ng/ml (0.6 nM)
**Electrochemical Sensing**	PVD oxidation on carbon electrodes coated with electrochemically generated graphene functionalized with gold nanoparticles (Gandouzi et al., 2018)	A linear range of 1-100 μM pyoverdine in PBS. Detected 25 μM pyoverdine in serum, saliva and tap water matrices.	LOD: 333.33 nM
	Pyoverdine sensing with aptamers (this report)	A dynamic range over three log units from 5 - 1000 nM. Specific for pyoverdine over other siderophores - enterobactin and pseudobactin	LOD: 1.3 nM

Legend: A summary is given of the current methods for detecting pyoverdine. Some (colorimetric, fluorescence) require first extracting the pyoverdine to separate it from many other compounds that contribute to background noise.

Table S2. Sequences of aptamers

15THR1A	GGTTGGTGTGGTTGG
21COD1A	TGGGTCGGGAGGGAATGCGGG
32THR4A	GGTAGGGCAGGTTGGGTGTTTTCACTTTTGGG
55PYO2A	CTAGGAATCAGGAGCGAATGTTAGAGTATTTGGATAGTATGTTTATT GTATCGGC
55PYO4A	GATCGGAATGGGAGCGA ATGAG GAGGGAGCGCGGACATGGTGCAGCAGCCCGACG
BRC	GAGACGGUCGGGUCCAGAUAUUCGUAUCUGUCGAGUAGAGUGUGGGC UC

Table S3. Sequences of other nucleic acids used in DNA SELEX

Oligo 5615 5' primer	GCCTGTTGTGAGCCTCCTGTCGAA
Oligo 5617 3' primer	GGGAGACAAGAATAAACGCTC
Oligo 5625 5' primer complement	TTCGACAGGAGGCTCACAACAGGC
Oligo 5636 55PYO2A with primer binding region	GCCTGTTGTGAGCCTCCTGTCGAACTAGGAATCAGGAGC GAATGTTAGAGTATTTGGATAGTATGTTTATTGTATCGGCG AGCGTTTATTCTTGTCTCCC
Oligo 5637 Control oligonucleotide	GCCTGTTGTGAGCCTCCTGTCGAAGTATGCGATAGGAGC GAATGATATATATCAAGTTACCACACGGATGTACTTGCTTG AGCGTTTATTCTTGTCTCCC
Oligo 5685 55PYO4A with primer binding region	GCCTGTTGTGAGCCTCCTGTCGAAGATCGGAATGGGAGC GAATGAGGAGGGAGCGCGGACATGGTGCAGCAGCCCGA CGGAGCGTTTATT

Table S4: Selection scheme for DNA aptamers that bind PVD-Pf5.

Round	ssDNA: PVD-Pf5	Target	Length of capture oligo (nt)	Incubation temperature
1	1:1	Ferric PVD-Pf5	6	4
2	2:1	Ferric PVD-Pf5	6	4
3	3:1	Ferric PVD-Pf5	7	4
Counter selection	1:1	Ferric enterobactin and Ferric ornibactin	7	4
4	5:1	Ferric PVD-Pf5	8	4
5	6:1	Ferric PVD-Pf5	8	23
6	7:1	Ferric PVD-Pf5	8	23
Counter selection	1:1	Ferric enterobactin and Ferric ornibactin	8	23
7	8:1	Ferric PVD-Pf5	8	23
8	10:1	Ferric PVD-Pf5	9	23
9	10:1	Ferric PVD-Pf5	9	23
10	10:1	Ferric PVD-Pf5	9	23

Legend: Ferric-enterobactin and ferric-ornibactin were used as counter selection targets. Selection was performed in SSMA2.

FIGURES

Figure S1. NextGEN sequencing data

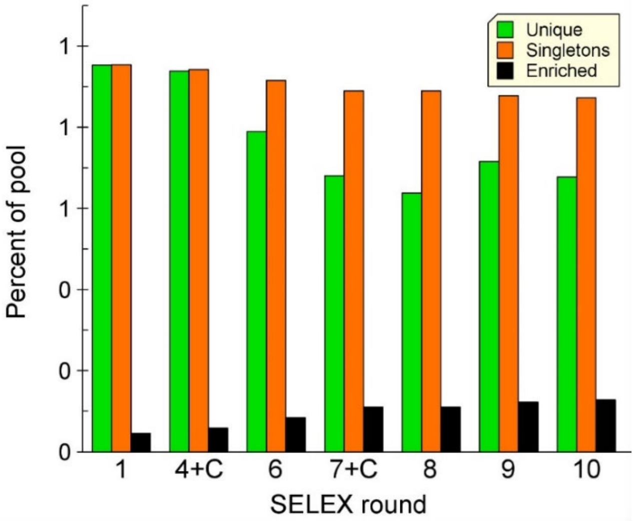

Legend: Next gen sequencing data from DNA SELEX showed downward trends of the unique fraction (number of species in pool divided by pool size) and singletons (species present only once in the database) and a rise in the enriched species (species present more than once in the database).

Figure S2: Screening for aptamers

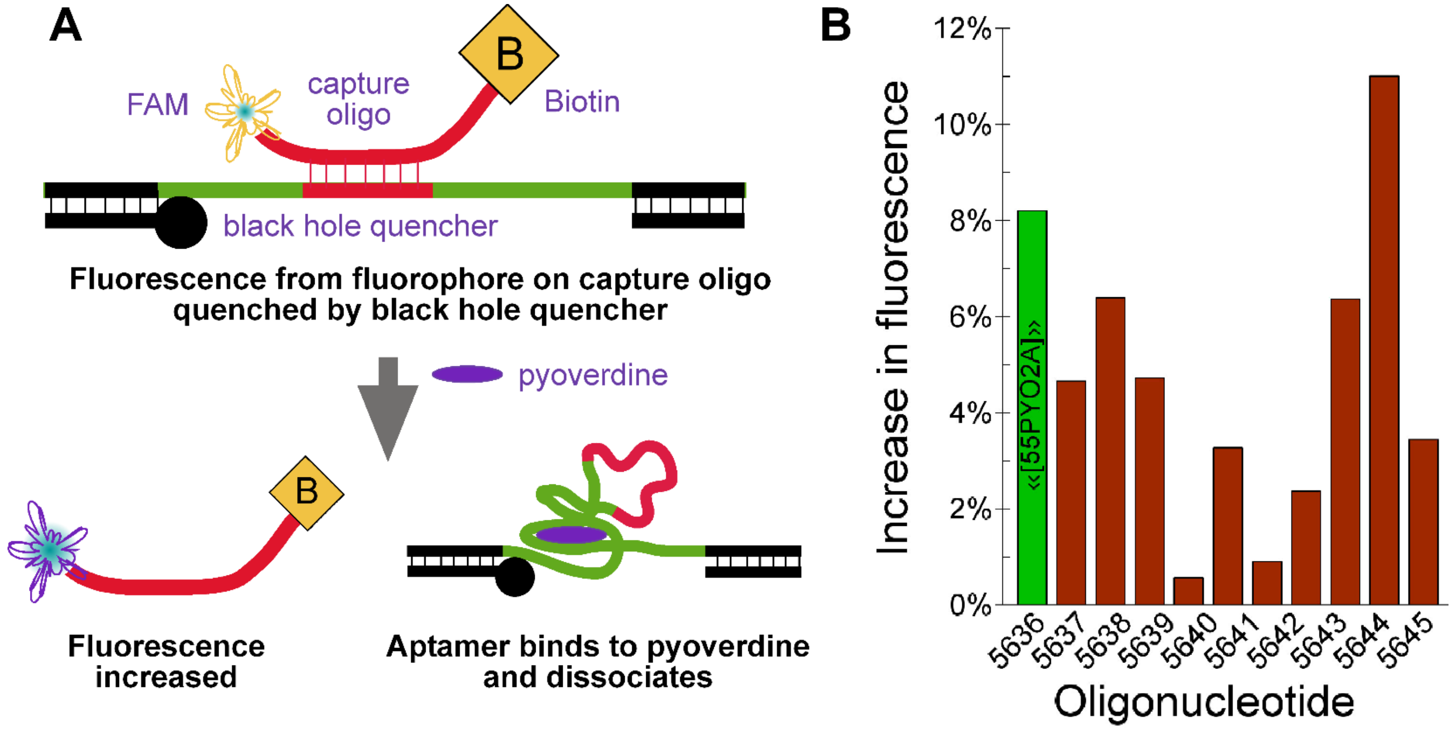

LEGEND: Aptamer screening by fluorescence quenching assay **A)** ssDNA aptamer candidates were hybridized with FAM (6-Carboxyfluorescein) and biotin-labeled capture oligo and black hole quencher labeled blocking oligo on 5' end and blocking oligo on 3' end. FAM fluorescence was quenched by the black hole quencher in the hybridized complex (capture oligo-aptamer-blocking oligos). After binding with pyoverdine the aptamer dissociates from complex and fluorescence increases from the fluorophore attached to the capture oligo. This assay was performed in streptavidin coated microwells by which the biotinylated capture oligo was held to the surface. The depiction in A is based on Fig. 1 for aptamer screening in (Zhao et al., 2019). **B)** Binding of 40nM candidate aptamers with 800nM pyoverdine. Candidate 5636 contains the sequence of 55PYO2A

Figure S3: The structure of PVD-Pf5

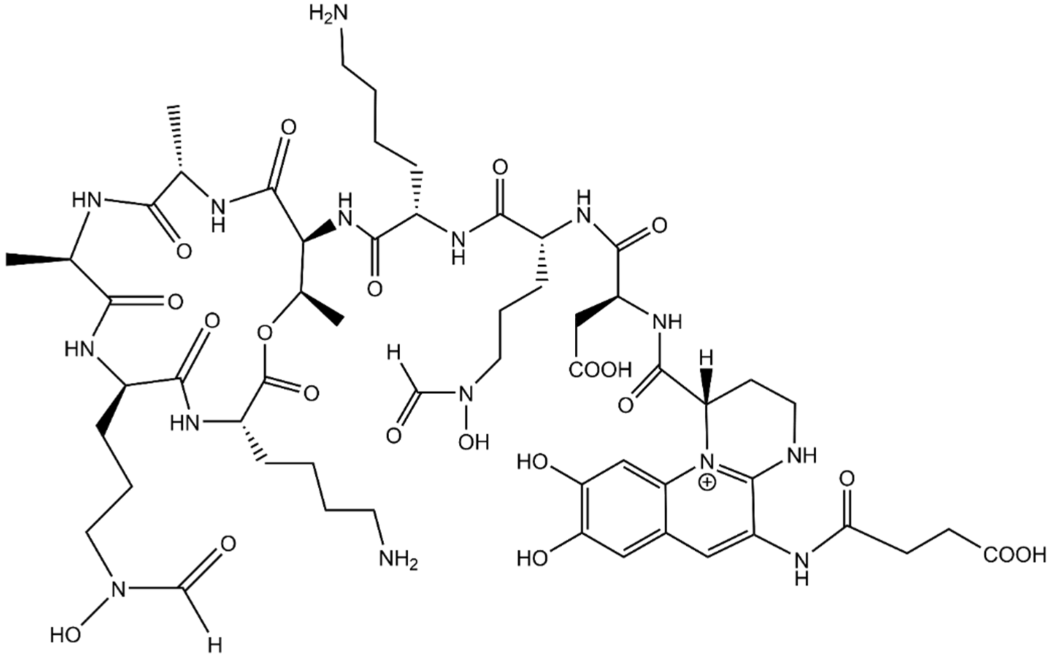

Legend: The structure of PVD-Pf5 is shown (Hartney et al., 2013)

Citations

Cueva, A. R., Pham, O., Diaby, A., Fleming, D., Rumbaugh, K. P. & Fernandes, G. E. 2020. Pyoverdine Assay for Rapid and Early Detection of Pseudomonas aeruginosa in Burn Wounds. *ACS Applied Bio Materials,* 3**,** 5350-5356.

Gandouzi, I., Tertis, M., Cernat, A., Bakhrouf, A., Coros, M., Pruneanu, S. & Cristea, C. 2018. Sensitive detection of pyoverdine with an electrochemical sensor based on electrochemically generated graphene functionalized with gold nanoparticles. *Bioelectrochemistry,* 120**,** 94-103.

Hartney, S. L., Mazurier, S., Girard, M. K., Mehnaz, S., Davis, E. W., 2nd, Gross, H., Lemanceau, P. & Loper, J. E. 2013. Ferric-pyoverdine recognition by Fpv outer membrane proteins of Pseudomonas protegens Pf-5. *Journal of bacteriology,* 195**,** 765-776.

Louden, B. C., Haarmann, D. & Lynne, A. M. 2011. Use of Blue Agar CAS Assay for Siderophore Detection. *J Microbiol Biol Educ,* 12**,** 51-3.

Zhao, M., Li, W., Liu, K., Li, H. & Lan, X. 2019. C4-HSL aptamers for blocking qurom sensing and inhibiting biofilm formation in Pseudomonas aeruginosa and its structure prediction and analysis. *PloS one,* 14**,** e0212041.
